# Editorial: Genetics, genomics, and breeding of edible mushrooms in Asia

**DOI:** 10.3389/fmicb.2024.1390845

**Published:** 2024-03-18

**Authors:** Chenyang Huang, Vikineswary Sabaratnam, Tadanori Aimi

**Affiliations:** ^1^Institute of Agricultural Resources and Regional Planning, Chinese Academy of Agricultural Sciences, Beijing, China; ^2^State Key Laboratory of Efficient Utilization of Arid and Semi-arid Arable Land in Northern China, Beijing, China; ^3^Key Laboratory of Microbial Resources, Ministry of Agriculture and Rural Affairs, Beijing, China; ^4^Mushroom Research Centre, Institute of Biological Sciences, Faculty of Science, Universiti Malaya, Kuala Lumpur, Malaysia; ^5^Faculty of Agriculture, Tottori University, Tottori, Japan

**Keywords:** edible mushrooms, Asia, genetics, genomics, breeding

Mushrooms are unique, as described by Chang and Miles (1989) in the following quote: “Without leaves, without buds, without flowers, yet, they form fruit; as a food, as a tonic, as a medicine, the entire creation is precious”. Influenced by different histories and cultures, Asians prefer to eat edible mushrooms. Consequently, in Asia, both the scale of production and the level of cultivation technology used are very high. The edible mushroom industry in Asia accounts for more than 85% of the world's total production. Research on the genetics, genomics, and breeding of edible mushrooms in Asia is growing rapidly. The theme of this Research Topic is to gather the progress of research conducted on Asian edible mushrooms. It is gratifying that the research on Asian edible mushrooms is conducted in an all-round manner.

## In terms of species

Asians not only eat and study button mushrooms, there are more species, but also currently commercially cultivated nearly 100 species of mushrooms. Eight species were studied in this Research Topic: the bulk species are *Pleurotus ostreatus* (Liu et al.), *Pleurotus cornucopiae* (Qi et al.), *Auricularia heimuer* (Qian et al.), and *Auricularia cornea* (Ma et al.); the rare species are morels (*Morchella* spp.) (Chen et al.) and *Hymenopellis radicata* (Cao et al.); the medicinal species are *Ganoderma sichuanense* (Li et al.) and *Inonotus hispidus* (Wang et al.).

## From the perspective of the entire industrial chain

This Research Topic consists of five parts. 1. The first part focuses on genomics. Wang et al. present works on genomic comparison between two *Inonotus hispidus* strains isolated from growing in different tree species. Comparative genomics showed that the coding genes and the total number of genes annotated in different databases of *Fraxinus mandshurica* were higher than that of *Morus alba*. Ma et al. present works on high-quality genome assembly and multi-omics analysis of the pigment synthesis pathway in *Auricularia cornea*. The results showed that there were numerous inversions and translocations between homologous regions of white/purple *A. cornea*. The purple strain synthesized pigment via the shikimate pathway. Cao et al. present works on the population genetic structure of *Hymenopellis radicata* germplasm resources based on genome re-sequencing. 2. The second part deals with development and regulation. Qi et al. present works on milR20 that negatively regulates the development of fruit bodies in *Pleurotus cornucopiae*. The results showed that the function of milR20, which targeted pheromone A receptor *g8971*, was involved in the MAPK signaling pathway. 3. The third part focuses on breeding, Qian et al. present works on interspecies hybridization between *Auricularia cornea* cv. Yu Muer and *Auricularia heimuer* cv. Bai Muer through protoplast fusion. The hybrids and their parents showed significant differences in their colony morphology. Yellowish-white primordia were obtained from two hybrids. In my personal opinion, the genetic stability of the hybrids merits further attention. 4. The fourth part deals with the prevention and control of disease and pests. Li et al. present works on the characterization and fungicide sensitivity of *Trichoderma* species causing green mold of *Ganoderma sichuanense* in China. The results showed that Prochloraz manganese showed the best performance against most *Trichoderma* spp. Liu et al. present works on a lectin gene that is involved in the defense of *Pleurotus ostreatus* against the mite predator *Tyrophagus putrescentiae*. The findings shed light on the molecular mechanisms of *P. ostreatus'* defense against the mite predator. 5. The fifth part discusses continuous cropping obstacles. In response to the hottest species of morels in China, the most complex problem of continuous cropping obstacles is proposed to solve the problem. Chen et al. present works on how dazomet changes microbial communities and improves morel mushroom yield under continuous cropping. The results showed that dazomet improves morel mushroom yield under continuous cropping.

Overall, the research on Asian edible mushrooms has entered a period of full-scale outbreak, and we suggest that more and better research will continue to emerge.

At present, this Research Topic is a microcosm of the research on Asian edible mushrooms. In the future, this Research Topic will be a testimony to it.

## Author contributions

CH: Conceptualization, Funding acquisition, Validation, Writing—original draft, Writing—review & editing. VS: Validation, Writing—review & editing. TA: Writing—review & editing.
